# Association of Obesity with SARS-CoV-2 and Its Relationship with the Humoral Response Prior to Vaccination in the State of Mexico: A Cross-Sectional Study

**DOI:** 10.3390/diagnostics13162630

**Published:** 2023-08-09

**Authors:** Daniel Montes-Herrera, José Esteban Muñoz-Medina, Larissa Fernandes-Matano, Angel Gustavo Salas-Lais, Ma. De Los Ángeles Hernández-Cueto, Clara Esperanza Santacruz-Tinoco, Irma Eloisa Monroy-Muñoz, Javier Angeles-Martínez

**Affiliations:** 1 Central Epidemiology Laboratory, Mexican Social Security Institute, Mexico City 02990, Mexico; danielmontes_1990@hotmail.com (D.M.-H.); salas_lais@yahoo.com.mx (A.G.S.-L.);; 2 Quality of Supplies and Specialized Laboratories Coordination, Mexican Social Security Institute, Mexico City 07760, Mexico; eban10@hotmail.com (J.E.M.-M.);; 3 Specialized Laboratories Division, Mexican Social Security Institute, Mexico City 07760, Mexico; cest03b@gmail.com; 4 Reproductive and Perinatal Health Research Department, National Institute of Perinatology, Mexico City 11000, Mexico; irmae4901@gmail.com

**Keywords:** obesity, SARS-CoV-2, anti-S1/S2 IgG, symptomatic, asymptomatic

## Abstract

Obesity is associated with an increased risk of contracting infections. This study aimed to estimate the risk of COVID-19 infection associated with obesity and to assess its role in the specific antibody response against SARS-CoV-2 in 2021. This study included 980 participants from the State of Mexico who participated in a serological survey where they were tested for SARS-CoV-2 IgG anti-S1/S2 and anti-RBD antibodies and asked for height, weight, and previous infection data via a questionnaire. Of the cohort of 980 participants, 451 (46.02%) were seropositive at the time of recruitment (45.2% symptomatic and 54.8% asymptomatic). The risk of SARS-CoV-2 infection with obesity was 2.18 (95% CI: 1.51–3.16), 2.58 (95% CI: 1.63–4.09), and 1.88 (95% CI: 1.18–2.98) for seropositive, asymptomatic, and symptomatic individuals, respectively, compared to those with normal weight. Anti-S1/S2 and anti-RBD IgG antibodies tended to be higher in overweight and obese participants in the seropositive group and stratified by different obesity classes. Additionally, there was a positive correlation between anti-S1/S2 and anti-RBD IgG antibodies and BMI in both men and women in the seropositive group. Obesity is an independent risk factor for SARS-CoV-2 infection when adjusted for confounding variables; however, the relationship between BMI and anti-S1/S2 and anti-RBD IgG antibody levels differed markedly in the presence or absence of symptoms.

## 1. Introduction

At the end of 2019, a new infectious disease called coronavirus disease (COVID-19) severely affected human health and daily living activities. Due to its high transmissibility, on March 11, 2020, it was listed as a pandemic by the World Health Organization (WHO). Populations with the highest risk in the SARS-CoV-2 pandemic include people with comorbidities such as obesity, diabetes, and cardiac, pulmonary, or neurological diseases [1,2]. Prompt and timely identification of infected individuals is necessary to control transmission and contain the risk of spread of SARS-CoV-2, regardless of the presence or absence of COVID-19 symptoms [3].

The gold standard test for SARS-CoV-2 diagnosis is nucleic acid amplification [4], and serological tests have been used successfully for conducting epidemiological serological surveys, investigating the antibodies responses against SARS-CoV-2 infection, and evaluating the infection prevalence in the population [5]. Serological tests must allow adequate interpretation and application of the results; such tests must accurately identify patients with a COVID-19 infection background and ought to allow the correct prediction of the protective immunity acquired by previous infection or vaccination [6,7,8,9]. The evaluation of different types of SARS-CoV-2 antibody detection tests has demonstrated that the use of assays directed against IgG anti-S1/S2 leads to high diagnostic accuracy and confirmed that detected antibody concentrations are strongly associated with the intensity of neutralizing antibodies regardless of the assay [10,11,12,13]. However, people with comorbidities showed different degrees of immune response to infection or immunization [14].

On the other hand, obesity is one of the most prevalent comorbidities worldwide, and is an important risk factor for several non-communicable diseases such as diabetes mellitus, myocardial infarction, and non-alcoholic fatty liver disease [15]. Furthermore, people with obesity who develop COVID-19 have a higher rate of hospitalization, more risk of severe progression, and worse clinical outcomes [16]. In addition to this, overweight and obesity have a high prevalence worldwide, in 2016, 39% of adults over 18 years of age developed overweight and 13% obesity [17]. In Mexico, the situation is very similar, since in 2018 it was reported that 39.1% of adults over 20 years of age developed overweight and 36.1% obesity [18]. This fact became reflected in the data reported for COVID-19 in our country, as obesity (10.48%), arterial hypertension (12.65%), and diabetes (9.50%) were the main comorbidities found in patients with COVID-19 [1].

Therefore, the first objective of this study was to estimate the risk of SARS-CoV-2 infection associated with obesity, both in symptomatic and asymptomatic individuals in a cohort of adults before their first dose of vaccine against COVID-19. Our second objective was to assess the role of obesity in IgG anti-S1/S2 and specific neutralizing anti-RBD antibody responses against SARS-CoV-2.

## 2. Materials and Methods

### 2.1. Study Design and Population

This is a cross-sectional study performed to evaluate the immunological status of the Mexican population before the application of any vaccination scheme against SARS-CoV-2. Participants were recruited from mass vaccination centers located in five municipalities of the State of Mexico between 14 May and 27 July 2021. Participation was voluntary and without restrictions. The inclusion criteria of the participants were: adult participants without previous application of any vaccination scheme against COVID-19 and who agreed to sign the informed consent form. Exclusion criteria were the presence of signs or symptoms of respiratory disease, and refusal to sign the informed consent form.

### 2.2. Demographic Variables

Data extracted included baseline demographic information, such as sex (women and men), age (years), smoking history, alcohol consumption, drug use, medication use, hypertension (HT), type 2 diabetes (T2D), autoimmune disease (AD), other respiratory disease (ORD), heart disease (HD), date of diagnostics for those who reported a previous infection (COVID-19 confirmed case was defined as a positive RT–qPCR test, antigen test, or antibody test), and the number of days elapsed from the onset of the symptoms until the date of inclusion in the study. The data were processed and analyzed without personal identifiers to maintain the participant’s confidentiality.

### 2.3. Anthropometric Measurements

The measurements included participant weight (kg) and height (m). Body mass index (BMI) was calculated using the following formula: BMI = weight (kg)/height^2^ (m). The participants were classified by BMI as normal weight (BMI < 25 kg/m^2^), overweight (BMI 25–29.9 kg/m^2^), and obese (BMI ≥ 30 kg/m^2^). In addition, the subjects were classified by obesity class using the WHO reference ranges: class I or mild obesity, BMI 30–34.9 kg/m^2^; class II or moderate obesity, BMI 35–39.9 kg/m^2^; and class III or morbid obesity, BMI ≥ 40 kg/m^2^.

### 2.4. Detection of IgG Antibodies against S1/S2 Antigens of SARS-CoV-2

Blood samples were obtained in 5 mL BD Vacutainer tubes with separator gel. After collection, all the samples were centrifuged (10 min/3500 rpm) and sent under refrigeration conditions to the IMSS Central Epidemiology Laboratory (LCE) in category B biological samples packaging (UN 3373) following the recommendations of the International Air Transport Association (IATA) [19].

The IgG antibodies detection was carried out using 200 µL of serum and the LIAISON^®^ SARS-CoV-2 S1/S2 IgG kit (DiaSorin, Saluggia, Italy, cat. 311450) [10]. The test was conducted in LIAISON XL equipment following the manufacturer’s instructions. Negative and positive controls were used to validate the results. The cut-off values were as follows: negative < 15 AU/mL and positive > 15 AU/mL.

### 2.5. Neutralizing Activity of Antibodies against the RBD Antigen of SARS-CoV-2

The anti-RBD antibodies neutralizing activity was evaluated using the SARS-CoV-2 Surrogate Virus Neutralization Test ELISA kit (GenScript, Piscataway, NJ, USA, Cat. L00847) [11]. The kit contains the ACE2 cell surface receptor immobilized in 96-well plates and includes an analog of the receptor binding domain labeled with HRP (HRP-RBD), which was previously incubated with the sera to be analyzed. Serum-neutralizing antibodies bind to HRP-RBD and block its interaction with the ACE2 cellular receptor. The test was performed following the manufacturer’s instructions, and negative and positive controls were used to validate the results. The cut-off values were as follows: negative < 30% inhibition and positive > 30% inhibition.

### 2.6. Statistical Analysis

Descriptive statistics were used to evaluate the frequency and percentage. Continuous data are presented as medians with interquartile ranges (IQRs). The distribution normality was tested using the Kolmogorov–Smirnov test. The differences between continuous variables in the groups were evaluated with the Mann–Whitney U test and the Kruskal–Wallis test. Nominal variables were analyzed using the chi-square test. The analysis was performed with the statistical package SPSS version 20.0 (SPSS, Chicago, IL, USA). Differences were considered significant when *p* < 0.05. To evaluate the association of obesity and obesity class with SARS-CoV-2 infection, a logistic regression model adjusted for age, sex, smoking status, and alcohol use was developed. Statistical significance was evaluated using 95% confidence intervals and an 80% statistical power. Bonferroni correction was used for multiple tests due to the higher risk of type I error (0.05 divided by 5, *p* < 0.01). To examine the relationships between the variables, Spearman’s correlation analysis was performed. GraphPad Prism version 8 (GraphPad Software, San Diego, CA, USA) was used to make all graphs.

## 3. Results

Samples from 980 participants were collected in five municipalities of the State of Mexico (Table 1). Using the collected information and the anti-S1/S2 IgG antibodies and anti-RBD neutralizing antibodies tests results, the participants were classified into three groups: (i) seronegative: participants who were negative for anti-S1/S2 and anti-RBD IgG and who also denied previous contact with the virus; (ii) symptomatic: participants who reported a prior SARS-CoV-2 infection and were positive for anti-S1/S2 and anti-RBD IgG (serologically negative participants in this group were excluded from antibody level analysis); and (iii) asymptomatic: participants who reported no prior SARS-CoV-2 infection but tested positive for anti-S1/S2 and anti-RBD IgG.

Overall, the prevalence of participants with prior SARS-CoV-2 infection was 46.02%, of whom 54.8% were asymptomatic and 45.2% presented symptoms. The distribution by sex showed a higher percentage of asymptomatic women (64.4%) than men (35.6%). The median BMI in participants with prior SARS-CoV-2 infection was 28.3 kg/m^2^. Significant differences were observed in comorbidities between the previous SARS-CoV-2 infected participants and the seronegative participants: class I obesity (25.9% vs. 19.1%, *p* = 0.0007), class II obesity (6.7% vs. 3.0%, *p* = 0.0008) and mild tobacco use (13.1% vs. 7.1%, *p* = 0.0058). More than half of the participants with a history of SARS-CoV-2 infection (symptomatic or asymptomatic) were older than 40 years of age.

The asymptomatic participants had a higher prevalence of class I obesity (27.1% vs. 19.1%, *p* = 0.0004), class II obesity (6.1% vs. 3.0%, *p* = 0.0040), and mild tobacco consumption (13.4% vs. 7.1%, *p* = 0.0150) compared with seronegative participants. Finally, the symptomatic group presented a higher prevalence of class II obesity (7.4% vs. 3.0%, *p* = 0.0080) and mild tobacco use (12.7% vs. 7.1%, *p* = 0.0357). When performing a within-group analysis of symptomatic or asymptomatic individuals, no significant differences were found.

### 3.1. BMI Association with Prior SARS-CoV-2 Infection

In the study population, obesity was the most frequent comorbidity (29.3%). When stratified by group, the highest percentage was found in the group with previous SARS-CoV-2 infection (35.5%), differing significantly (*p* < 0.00001) from the seronegative group. A similar result was found when symptomatic (34.3% vs. 24%, *p* = 0.0144) and asymptomatic (36.4% vs. 24%, *p* < 0.00001) groups were compared. In our study population, obesity is associated with SARS-CoV-2 infection. Participants with obesity had a risk of infection 2.11 times higher than those without obesity (OR = 2.11, 95% CI: 1.49–3.01, *p* < 0.0001); the association remained when participants were stratified into symptomatic (OR = 1.77, *p* = 0.011) and asymptomatic (OR = 2.49, *p* < 0.0001) groups. Furthermore, when compared by obesity class, participants with class I (OR = 1.94, *p* < 0.0001) and class II (OR = 3.15, *p* = 0.0010) obesity showed a higher risk of infection.

An association of previous SARS-CoV-2 infection with class I (OR = 2.33, *p* < 0.0001) and class II (OR = 3.30, *p* = 0.0030) obesity in the group of asymptomatic participants was found; in the group of symptomatic participants, there was only an association with class II obesity (OR = 3.01, *p* = 0.0050) (Table 2). The same analysis was conducted after adjusting for age, sex, smoking status, and alcohol consumption, where people with obesity had a higher risk of infection (OR = 2.18, *p* < 0.0001).

### 3.2. BMI Influence over Anti-S1/S2 and Anti-RBD IgG Antibodies Response

To estimate the general antibody response against SARS-CoV-2 in the participant’s serum before starting their vaccination scheme, the presence of anti-S1/S2 and anti-RBD IgG antibodies was analyzed. Anti-S1/S2 IgG antibodies and anti-RBD neutralizing activity were detected in 176 of the 204 participants with a history of SARS-CoV-2 infection. A total of 225 participants were considered asymptomatic, as they reported no previous contact with the virus but tested positive for IgG anti-S1/S2 and anti-RBD antibodies. 

**Figure 1:**
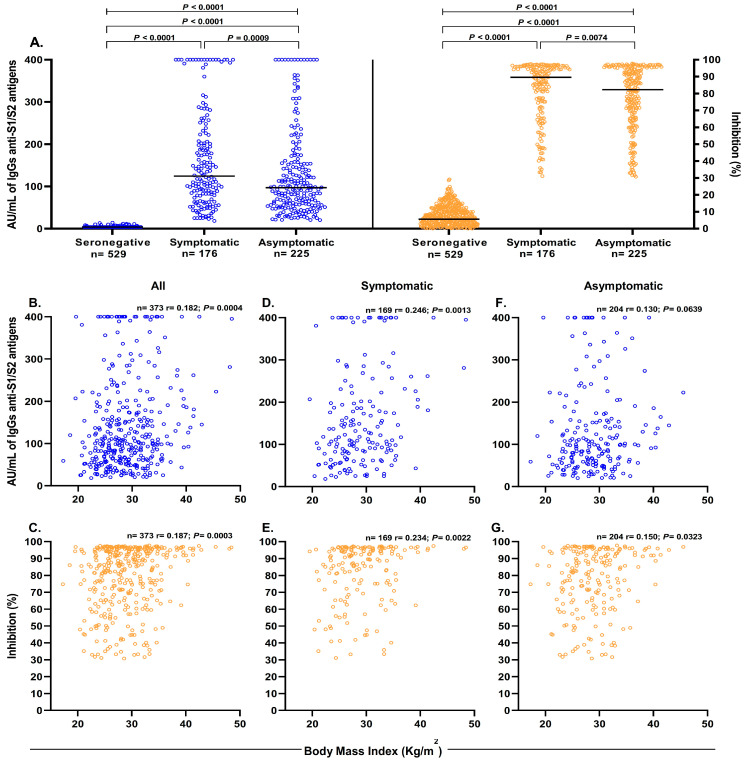
Anti-S1/S2 IgG antibodies levels and anti-RBD neutralizing antibodies positively correlate with BMI. (**A**) Levels of anti-S1/S2 IgG antibodies (blue) and anti-RBD neutralizing antibodies (orange) in symptomatic, asymptomatic, and seronegative participants. Points, individuals; bars medium; comparisons using the Mann–Whitney U or Kruskal–Wallis tests, as appropriate. Correlation between BMI and anti-S1/S2 IgG antibody levels in all seropositive (**B**), symptomatic (**D**), and asymptomatic (**F**) participants. Correlation between BMI and anti-RBD neutralizing antibodies in all seropositive (**C**), symptomatic (**E**), and asymptomatic (**G**) participants. r denotes Spearman’s correlation coefficient.

Compared to the seronegative group (median: 3.8 AU/mL), the seropositive groups (symptomatic and asymptomatic) had higher levels of anti-S1/S2 IgG antibodies (median: 124.5 AU/mL and 97.2 AU/mL, respectively). Within the group of seropositive participants, compared with the asymptomatic participants, the symptomatic participants had higher levels of anti-S1/S2 IgG and anti-RBD neutralizing antibodies (*p* < 0.05) (Figure 1A).

The correlation between BMI and anti-S1/S2 IgG antibodies level and the amount of neutralizing anti-RBD antibodies in our cohort of participants with a history of SARS-CoV-2 infection was analyzed. A general comparison of the participants showed a strong positive correlation between BMI and anti-S1/S2 IgG antibodies level (Figure 1B). More importantly, there was a positive correlation between BMI and the amount of anti-RBD neutralizing antibodies (Figure 1C). Similarly, a strong positive correlation was observed between BMI and the levels of anti-S1/S2 IgG antibodies and anti-RBD neutralizing antibodies in the group of symptomatic participants (Figure 1D,E); in the group of asymptomatic patients, there was a weaker correlation between BMI and levels of anti-RBD neutralizing antibodies (Figure 1G).

**Figure 2:**
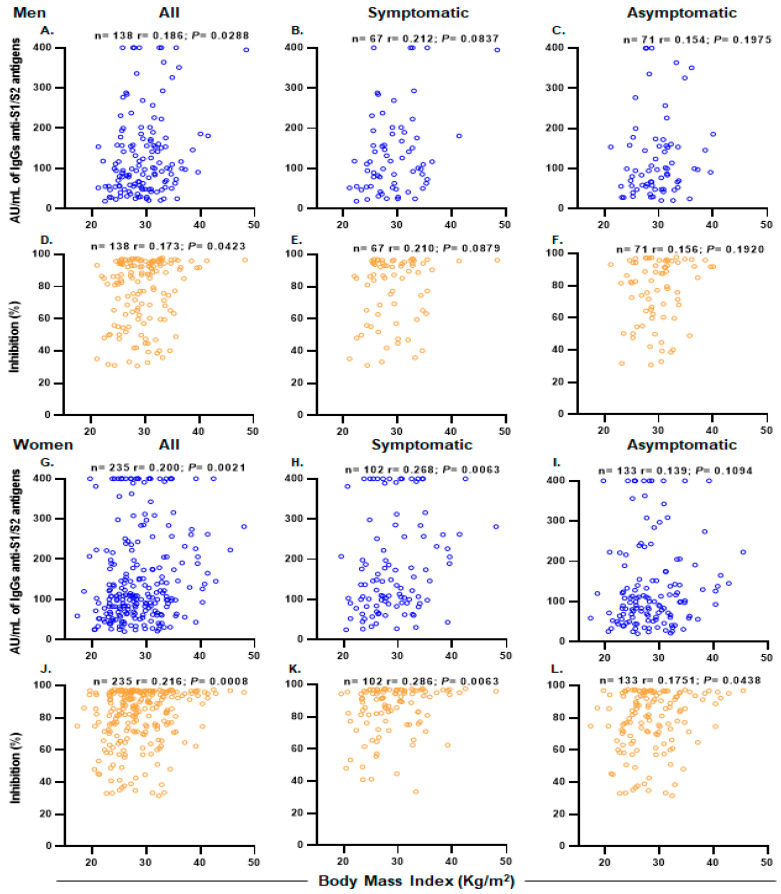
Correlation between BMI and antibody responses in seropositive participants stratified by sex. Comparison between BMI and anti-S1/S2 IgG antibodies and anti-RBD antibody levels in (**A**,**D**) seropositive (*n* = 138), (**B**,**E**) symptomatic (*n* = 67) and (**C**,**F**) asymptomatic (*n* = 71) men. Comparison between BMI and anti-S1/S2 IgG antibodies and anti-RBD antibody levels in (**G**,**J**) seropositives (*n* = 235), (**H**,**K**) symptomatic (*n* = 102) and (**I**,**L**) asymptomatic (*n* = 133) women. r denotes Spearman’s correlation coefficient.

In an analysis stratified by gender, we observed differences in the levels of anti-S1/S2 and anti-RBD IgG antibodies between men and women. We observed higher anti-S1/S2 IgG antibodies levels in women (median: 108.5 AU/mL) compared with men (median: 98.9 AU/mL, *p* = 0.0239) in symptomatic participants. Additionally, we observed higher anti-RBD IgG antibodies levels in women (median: 90.77%) compared with men (median: 86.81%, *p* = 0.0474) in symptomatic participants. In addition, in a correlation analysis in seropositive participants, BMI was correlated with higher levels of anti-S1/S2 IgG antibodies and anti-RBD neutralizing antibodies in women (r = 0.200, *p* = 0.0021 and r = 0.216, *p* = 0.0008, respectively) (Figure 2F,I). A mild correlation was also observed for men (r = 0.186, *p* = 0.0288 and r = 0.173, *p* = 0.0423, respectively) (Figure 2A,C).

### 3.3. Obesity and Other Comorbidities Influence the Anti-S1/S2 and Anti-RBD IgG Antibodies Response

**Figure 3:**
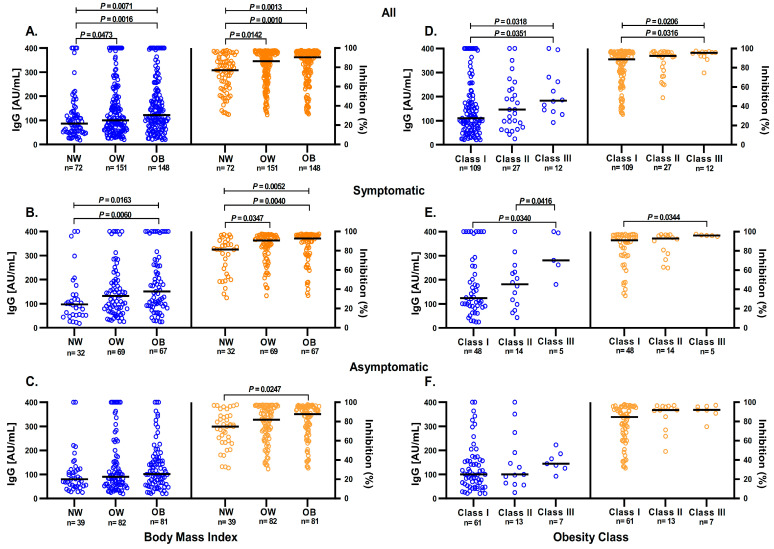
Anti-S1/S2 IgG antibodies levels and anti-RBD neutralizing antibodies in seropositive samples separated by BMI and obesity class. (**A**) Seropositive participants were stratified according to their BMI into normal weight (NW), overweight (OW), and obesity (OB), and compared their levels of anti-S1/S2 IgG antibodies and antibodies with anti-RBD neutralizing activity. (**B**) Comparison of anti-S1/S2 IgG antibody levels and anti-RBD antibodies in the symptomatic and (**C**) asymptomatic groups. (**D**) Comparison of anti-S1/S2 IgG antibodies and anti-RBD antibodies stratifying our seropositive group into obesity classes. (**E**) Comparison of anti-S1/S2 IgG antibodies and anti-RBD antibodies in the symptomatic and (**F**) asymptomatic groups. Points, individuals; bars medium; comparisons using the Mann–Whitney U or Kruskal–Wallis tests, as appropriate.

The study participants were stratified by normal weight, overweight, and obesity, as well as by obesity class (class I, class II, and class III). Seventy-two participants with normal weight, 151 with overweight, and 148 with obesity were identified (Figure 3). In comparison with normal-weight participants, overweight and obese participants had significantly higher levels of anti-S1/S2 IgG antibodies and anti-RBD neutralizing antibodies (Figure 3A). The difference in the anti-S1/S2 IgG antibodies level in symptomatic participants was maintained in people with obesity, and the difference in the anti-RBD antibodies level remained in overweight and obese people (Figure 3B). However, these differences were not observed in the anti-S1/S2 IgG antibodies level of asymptomatic participants, but remained for anti-RBD antibodies level when comparing participants with normal weight with those with obesity (Figure 3C).

Interestingly, in the population with a BMI ≥ 30 kg/m^2^ further stratified by obesity class, participants with class III obesity had higher levels of anti-S1/S2 IgG antibodies and anti-RBD antibodies than did participants with class I obesity (Figure 3D). Similarly, in the symptomatic group, the levels of anti-S1/S2 IgG antibodies and anti-RBD antibodies were higher in participants with class III obesity than in participants with class I obesity (Figure 3E); there were no statistically significant differences for the asymptomatic participants (Figure 3F).

**Figure 4:**
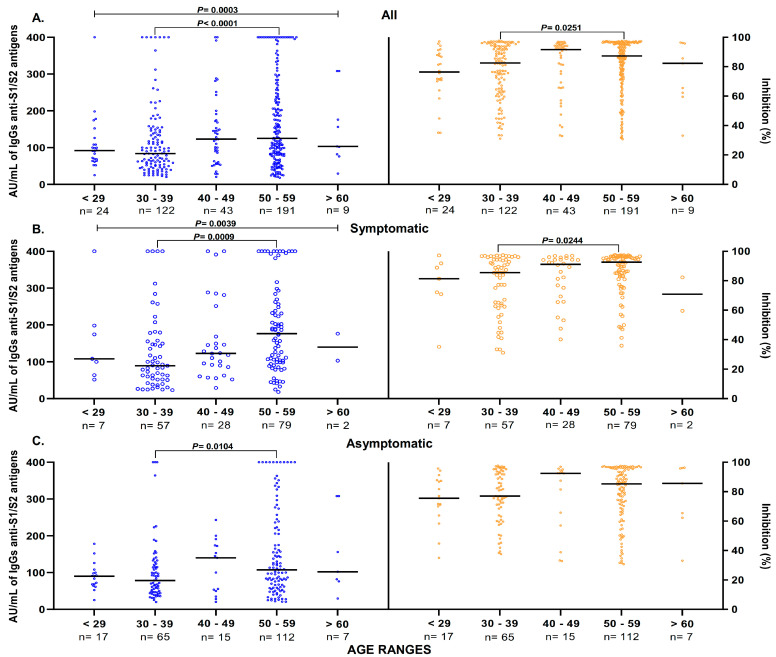
Anti-S1/S2 IgG antibodies and anti-RBD antibody levels in seropositive samples separated by age ranges. (**A**) Observed higher anti-S1/S2 IgG antibodies and anti-RBD antibody levels in the 50–59 years (median: 125 AU/mL, 87.26%) participants compared with the 30–39 years (median: 83.95 AU/mL, 82.57%) participants. (**B**) Analysis in symptomatic participants. Anti-S1/S2 IgG antibodies and anti-RBD antibody levels are higher in 50–59 years (median: 176 AU/mL, 92.65%) participants compared with the 30–39 years group (median: 89.60 AU/mL, 85.57%). (**C**) Analysis in asymptomatic group. Anti-S1/S2 IgG antibodies levels were significantly higher in the 50–59-year-old group compared to the 30–39-year-old group (median: 107.5 AU/mL vs. 78.6 AU/Ml, respectively). The black line represents the median anti-S1/S2 IgG antibodies and anti-RBD antibody levels for each group. The Mann–Whitney U test or Kruskal–Wallis test was used to evaluate differences across the groups. Values of *p* = 0.05 were taken as significant.

Based on the results, the influence of other variables, such as age, sex, and/or comorbidities, on the antibody response was assessed. The results showed significantly higher levels of anti-S1/S2 IgG antibodies in the 50–59-year-old group (*n* = 191) than in the 30–39-year-old group (*n* = 122) (median: 125 AU/mL vs. 91 AU/mL *p* < 0.0001) in seropositive participants. In this same group of participants, similar trends were observed when the percentage of inhibition was analyzed for the group aged between 50 and 59 years and the group aged between 30 and 39 years (median: 87.26% vs. 82.57%, *p* = 0.0251) (Figure 4A). According to these data, in symptomatic participants, the levels of anti-S1/S2 IgG antibodies and anti-RBD antibodies were higher in the group aged between 50 and 59 years (*p* = 0.0009 and *p* = 0.0244, respectively) than in the group aged between 30 and 39 years (Figure 4B). In the group of asymptomatic participants, those between 30 and 39 years of age had lower levels of anti-S1/S2 IgG antibodies than did those between 50 and 59 years of age (median: 78.6 AU/mL vs. 107.5 AU/mL, *p* = 0.0104) (Figure 4C).

**Figure 5:**
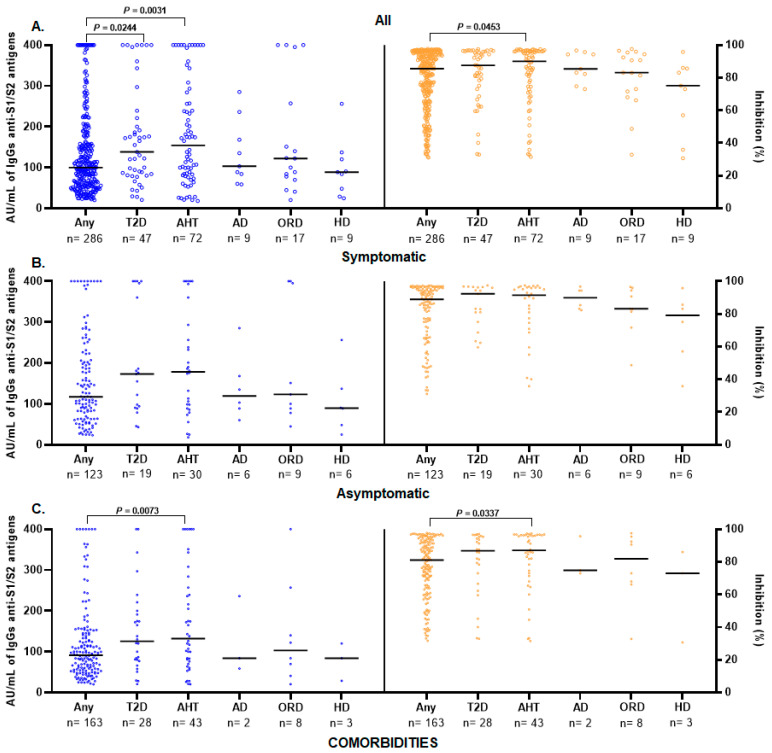
Comorbidities and antibody responses elicited in symptomatic and asymptomatic participants seropositive. (**A**) Seropositive participants with T2D have significantly higher anti-S1/S2 IgG antibodies levels than those without any comorbidity (median: 138 AU/mL vs. 99.85 AU/mL), as well as those with AHT (median: 154 AU/mL, 99.85 AU/mL, respectively), this significance is maintained with the anti-RBD antibodies levels in participants with AHT (median: 90.07%, 85.51%, respectively). (**B**) The analysis of the symptomatic group shows no significant differences. (**C**) In the asymptomatic group, we observed significantly elevated anti-S1/S2 IgG and anti-RBD antibody levels in the AHT (median: 132 AU/mL vs. 91 AU/mL and 87.02% vs. 81.15%, respectively) group compared to those without any comorbidity. The Mann–Whitney U test or Kruskal–Wallis test was used to evaluate differences across the groups. Values of *p* = 0.05 were taken as significant. Abbreviation: T2D, type 2 diabetes; AHT, arterial hypertension; AD, autoimmune disease; ORD, other respiratory diseases, and HD, heart disease.

Seropositive participants with T2D had a significantly higher anti-S1/S2 IgG antibodies level than those without comorbidities (median: 138 AU/mL vs. 99.85 AU/mL, *p* = 0.0309), as did those with hypertension (median: 154.0 AU/mL vs. 99.85 AU/mL, *p* = 0.0054); this significance remained for anti-RBD antibodies in hypertensive participants (median: 89.57% vs. 84.21%, *p* = 0.0434) (Figure 5A).

In the symptomatic group, there were no significant differences (Figure 5B); however, in the asymptomatic group, the levels of anti-S1/S2 IgG and anti-RBD antibodies were significantly higher in the hypertensive participants (median: 132 AU/mL vs. 91 AU/mL, *p* = 0.0073 and 87.02% vs. 81.15, *p* = 0.0337, respectively) than in those without comorbidities (Figure 5C).

### 3.4. Anti-S1/S2 IgG Antibodies and Anti-RBD Antibodies in Symptomatic Participants

**Figure 6:**
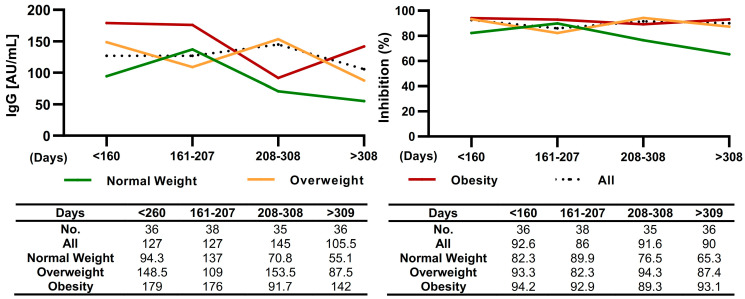
Anti-S1/S2 IgG antibodies and anti-RBD antibodies levels symptomatic participants over time. The *x*-axis indicates the timeline following the onset of symptoms divided into quartiles. The curves show the overall seropositivity of anti-S1/S2 IgG antibodies and anti-RBD antibodies in the groups with obesity (red line), overweight (orange line), normal weight (green line), and all participants (dashed black line).

The levels of IgG anti-S1/S2 antibodies and the neutralizing capacity of anti-RBD antibodies in participants with prior SARS-CoV-2 infection were analyzed by BMI. The time between the onset of symptoms and the sample collection was stratified into quartiles (Q1 < 160, Q2: 161–207, Q3: 208–308, Q4 > 309 days). The levels of IgG anti-S1/S2 antibodies and the neutralizing capacity of anti-RBD antibodies were elevated in the participants with a shorter time between the onset of symptoms and the time the sample was taken. In all groups, normal-weight, overweight, and obese, antibody levels decreased over time; however, the decrease in anti-S1/S2 and anti-RBD IgG antibody levels was greater in the normal-weight group than in the overweight and obese groups. Obese participants maintained elevated antibody levels for a longer period (Figure 6).

## 4. Discussion

In this study, we investigated the association between obesity and an increased risk of SARS-CoV-2 infection in symptomatic and asymptomatic participants. In particular, people with class I and class II obesity showed greater susceptibility to SARS-CoV-2 infection, even after adjusting for possible confounding factors (age, sex, smoking status, and alcohol use). This study is the first report that shows that the levels of anti-S1/S2 IgG antibodies and anti-RBD antibodies vary based on several factors, such as age, sex, comorbidities, symptomatic or asymptomatic disease, and BMI in the Mexican population.

In our cohort, when analyzing the levels of anti-S1/S2 IgG antibodies and anti-RBD antibodies, the symptomatic participants had significantly higher levels than the asymptomatic participants. This finding has been reported in other populations, where the levels of IgG against SARS-CoV-2 in the asymptomatic group were significantly lower than those in the symptomatic group in the acute phase [20,21]. Regarding the percentage of neutralization, a similar phenomenon occurs [22,23,24] in patients infected with another coronavirus, SARS-CoV [25]. Obesity is a risk factor for many non-communicable diseases, such as metabolic disorders and lung diseases, and can contribute to intensive care unit admissions and death among hospitalized patients with SARS-CoV-2 infection [15].

Frasca et al., 2021 observed that BMI is higher in positive individuals than in negative individuals (BMI: 27.7 vs. 23.5, respectively), observing a higher frequency of COVID-19 in individuals with a higher BMI [26]. Similar to previous findings, in this study, we found a higher prevalence of seropositive participants, compared with seronegative participants, with class I obesity (25.9% vs. 19.1%) and with class II obesity (6.7% vs. 3.0%). Furthermore, obesity has been reported to be a risk factor for SARS-CoV-2 infection. Popkin et al., in a 2020 meta-analysis, showed that people with obesity are at higher risk (OR = 1.46, *p* < 0.0001) than people without obesity [27] in a case–control study from South Korea (OR = 1.26 *p* < 0.05) [28], and in a retrospective analysis (0.06% vs. 0.01%, [OR = 6.13, *p* < 0.001]) using univariate analysis (OR = 2.20 [2.10–2.32], *p* < 0.05) and a logistic regression model [29], the same result was observed. Our results are consistent with these studies, as our unadjusted analysis showed that obesity is a risk factor associated with SARS-CoV-2 infection in seropositive, symptomatic, and asymptomatic subjects. To determine if this risk was present in the different classes of obesity, the sample was stratified into class I, II, and III obesity, and class I and II obesity continued to be a risk factor associated with COVID-19. For the adjusted analysis, obesity remained associated with seropositive, symptomatic, and asymptomatic participants. Interestingly, in the adjusted analysis, class I obesity was associated with seropositive and asymptomatic participants, and class II obesity was associated only with seropositive participants. Importantly, by stratifying the participants by degrees of obesity and symptomatic and asymptomatic seropositivity, a better perspective of the association between different variables and SARS-CoV-2 infection was obtained.

In this study, there was a discrete but statistically significant positive correlation between BMI and the magnitude of the IgG response and the neutralizing capacity of anti-RBD antibodies in symptomatic seropositive individuals but not in asymptomatic individuals. Similarly, a weak but significant correlation (r = 0.17, *p* = 0.002) has been reported between BMI and the IgG response against SARS-CoV-2 [21].

Regarding the differences when stratified by sex, the correlation between BMI and the levels of anti-S1/S2 IgG antibodies and anti-RBD antibodies was maintained in men and women. When analyzed by BMI group, in seropositive individuals, there were significantly higher levels of anti-S1/S2 IgG antibodies and anti-RBD antibodies in the obesity and overweight groups than in the normal-weight group. Other studies reported similar results because high levels of IgG against SARS-CoV-2 were associated with obesity. In addition, the neutralizing antibody titers were higher in individuals with COVID-19 with a BMI ≥ 30 kg/m^2^ than in those with a BMI < 25 kg/m^2^ [30,31]. This trend was maintained when adjusting for age, sex, ethnic origin, and time of onset of symptoms [21,30]. However, Nilles et al., 2021 did not find significant differences in viral neutralization or the activity of T cells when analyzing by BMI for individuals who suffered from COVID-19 [32]. However, this study included mostly asymptomatic individuals or individuals with mild disease (>98%), which limits the comparison of the effect of different degrees of obesity on COVID-19. In contrast, another study found a negative association between BMI and IgG values in patients infected with SARS-CoV-2 [27]. The role of elevated levels of IgG antibodies in the prognosis of COVID-19 should be investigated thoroughly because it has been reported that a large part of these antibodies in obese people are autoimmune, anti-malondialdehyde (a marker of oxidative stress and lipid peroxidation), and protein antigens derived from anti-adipocytes (markers of virus-induced cell death in obese adipose tissue) [27,33]. It has been suggested that in addition to antiviral efficacy, the antibody response could be associated with secondary organ damage mediated by antibodies [34].

Ko et al., 2020 found that asymptomatic and mild symptomatic groups were significantly younger than the pneumonia group (mean ages of 25.2, 30.9, and 65.7 years, respectively; *p* < 0.001) [35]; however, in our study, this difference was not observed. Hospitalization and COVID-19 severity are strongly associated with age and with increased IgG antibody responses against SARS-CoV-2 [31,36,37]. In the present study, older individuals (50–59 years) had a greater amount of antibodies IgG and neutralizing against SARS-CoV-2 than did younger individuals (30–39 years), findings that are similar to those reported by other authors [2,21,24,30,38,39]. This trend is interesting because a lower amount of anti-S1/S2 IgG antibodies and less neutralization are expected in older adults. After all, the immune system ages and does not generate a solid response to pathogens [2]. This may be due to the accumulation of chronic inflammatory conditions typical of aging because high values of IgA and IgG have been found in older subjects. In addition, there is a correlation between the concentrations of IgA and IgG and the serum concentrations of IL-6, a marker of inflammation and cofactor for the synthesis of immunoglobulins [40].

In this study, the median age of symptomatic individuals was 50 years. However, Karbiener et al. in 2021 found convalescent individuals with a mean age of 32 years [37], and Skorek et al. in 2021 found individuals with a mean age of 39 years [38]. Another important issue to study because of its close relationship with the prognosis of COVID-19 are the comorbidities, such as hypertension or diabetes, which are more frequent in older adults [2].

Various studies have indicated a higher prevalence of people with hypertension among patients with COVID-19 [41]; one study reported that 60% of their patients had a diagnosis of hypertension and an adjusted probability of having COVID-19 (OR = 2.53, *p* < 0.05) [29]. Interestingly, the data herein demonstrate that the percentage of people with hypertension who became ill with COVID-19 was slightly higher than that of uninfected people (16.9% vs. 16.6%). For individuals with T2D, the percentage was similar in both groups (10.9%). One study found that 31.5% of their patients had a diagnosis of diabetes mellitus and an increased risk of having COVID-19 (OR = 1.41, *p* < 0.05), suggesting that diabetes is an independent risk factor for COVID-19 [29]. Furthermore, in an adjusted analysis of the Italian population, both patients with hyperglycemia and patients with diabetes had a higher risk of severe disease than those without diabetes and with normoglycemia [42].

The levels of anti-S1/S2 and anti-RBD IgG antibodies in individuals with comorbidities were also measured, and seropositive individuals with hypertension and T2D had significantly higher levels of IgG-type antibodies than the participants without comorbidities. Regarding anti-RBD neutralizing activity, only participants with hypertension had a higher neutralization percentage. For symptomatic participants, differences between individuals with comorbidities and without comorbidities were not observed, and when analyzing the asymptomatic group, only significantly higher levels of anti-S1/S2 IgG antibodies and anti-RBD antibodies were observed in subjects with hypertension. We did not observe significant differences between the levels of anti-S1/S2 IgG and anti-RBD antibodies of the participants with any of the other comorbidities (AD, ORD, and HD) and those who did not have any comorbidities. Our finding corroborates the findings of a previous study, as no significant differences were found between the levels of IgG against SARS-CoV-2 in individuals with comorbidities such as cardiovascular disease and lung disease with those who did not [43]. However, in our study, the small number of participants with any of these comorbidities is a limitation and makes it difficult to conclude their effect on antibody levels. Similarly, it has been reported that the levels of IgG against SARS-CoV-2 are significantly higher in people with comorbidities of metabolic syndrome (T2D and hypertension) than in those without these comorbidities. IgG levels are significantly higher in patients with HbA1c ≥ 6.5% than in those with HbA1c < 5.7%, which may be related to higher levels of antibodies in individuals with T2D [30]. Additionally, an association was observed between individuals with hypertension and elevated IgG levels; however, this association becomes less strong after adjusting for age and sex [40]. Furthermore, it has been observed that the levels of IL-6 and D-dimer are significantly higher in hyperglycemic individuals than in normoglycemic individuals (*p* < 0.001), and this difference persists during hospitalization [42]. These findings suggest that people with comorbidities such as T2D and hypertension may also develop a more pronounced inflammatory response that leads to higher levels of antibodies and an increased risk of severe disease.

Sex hormones are an important biological factor that contributes to sex bias in the immune response and can influence disease severity in infections and autoimmunity [44]. In general, serum IgG and IgM levels are higher in women than in men [40]. Another study indicated, using a logistic regression model of workers’ ELISA results, that men have lower antibody values against SARS-CoV-2 than women [45]. This finding is similar to the results obtained in this study, in which there were significantly higher levels of anti-S1/S2 IgG antibodies in women than in men in the symptomatic group. In contrast, there are reports in which the average antibody titers were lower among women than men [31,38]. There are other studies in which there are no significant differences in serum IgG levels between sexes among individuals with mild disease, in recovery, and late stages [46] and among participants who had a second exposure to the viral antigen [47], but IgG levels in women increase significantly in the early phase of the disease, in severe cases [46], and in the first exposure to viral antigen [47], which is why it is important to take these variables into account.

Regarding prevalence, a retrospective cross-sectional study in the City of Monterrey found a seroprevalence 1.5 times higher in women than in men [48]. Similar to the above, in this study, 62.5% of the seropositive patients were women, and 37.5% were men. Despite this, various prevalence studies have shown that men with COVID-19 have a higher risk of progressing to a more advanced stage of the disease and even dying [49].

One advantage of this study is the large, enrolled cohort, in addition to the stratification carried out for different degrees of obesity, and types of participants, i.e., seropositive: symptomatic and asymptomatic, allowing us to observe different trends. However, the results may be limited by the fact that the data collected was self-reported and by the relatively small number of elderly participants (>60 years, *n* = 26). In addition, because a decrease in antibodies can lead to seroreversion (from seropositive to seronegative), some seronegative study participants have been previously infected.

## 5. Conclusions

In conclusion, in our study population, obesity is an independent risk factor for SARS-CoV-2 infection, both in symptomatic and asymptomatic infections. People with this health condition should be considered a vulnerable group. In general, we found elevated levels of anti-S1/S2 and anti-RBD IgG antibodies in people who are more likely to have a worse prognosis of COVID-19 resolution, such as people who are obese, older, and diagnosed with T2D or hypertension. However, women, who have been reported to have a better prognosis, had more anti-S1/S2 and anti-RBD IgG antibodies than men. Therefore, the present study raises questions about the role of IgG and neutralizing antibodies in the course and development of COVID-19.

## Figures and Tables

**Table 1:** Baseline demographics and clinical characteristics.

Characteristics	Prior COVID-19 *			Seronegative *n* = 529 (%)
	All *n* = 451 (%)	Symptomatic *n* = 204 (%)	Asymptomatic *n* = 247 (%)	
Age (years)	50 (36–54)	46 (37–53)	50 (35–55)	47 (36–54)
18–29	27 (5.9)	10 (4.9)	17 (6.8)	37 (6.9)
30–39	144 (31.9)	70 (34.3)	74 (29.9)	158 (29.9)
40–49	46 (10.2)	29 (14.2)	17 (6.8)	81 (15.3)
50–59	209 (46.3)	88 (43.1)	121 (48.9)	220 (41.6)
60–100	10 (2.2)	3 (1.5)	7 (2.8)	16 (3.0)
Weight (Kg)	74 (64–85)	75 (65–85)	74 (64–85)	70 (62–80) ^d^
Height (m)	1.61 (1.55–1.68)	1.62 (1.55–1.69)	1.60 (1.55–1.67)	1.60 (1.54–1.68) ^d^
BMI (Kg/m^2^) **	28.3 (25.4–32.0)	27.7 (25.1–32.5)	28.6 (25.5–31.7)	27.0 (24.4–30.1) ^d^
Normal weight	88 (19.5)	46 (22.5)	42 (17)	148 (27.9)
Overweight	169 (37.5)	79 (38.7)	90 (36.5)	219 (41.4)
Class I obesity	117 (25.9) ^a^	50 (24.5)	67 (27.1) ^c^	101 (19.1)
Class II obesity	30 (6.7) ^a^	15 (7.4) ^b^	15 (6.1) ^c^	16 (3.0)
Class III obesity	13 (2.9)	5 (2.5)	8 (3.2) ^c^	10 (1.9)
Sex				
Women	282 (62.5)	123 (60.3)	159 (64.4)	343 (64.8)
IMen	169 (37.5)	81 (39.7)	88 (35.6)	186 (35.2)
Comorbidities				
Diabetes mellitus	49 (10.9)	20 (9.8)	29 (11.7)	58 (10.9)
Hypertension	76 (16.9)	32 (15.7)	44 (17.8)	88 (16.6)
Autoimmune disease	13 (2.9)	8 (3.9)	5 (2.0)	16 (3.0)
Other respiratory disease	18 (3.9)	10 (4.9)	8 (3.2)	23 (4.3)
Heart disease	9 (1.9)	6 (2.9)	3 (1.2)	7 (1.3)
HIV	1 (0.2)	1 (0.5)	0 (0)	4 (0.7)
Smoking history				
Never	364 (80.7)	164 (80.4)	200 (80.9)	437 (82.6)
Mild	59 (13.1) ^a^	26 (12.7) ^b^	33 (13.4) ^c^	38 (7.1)
Moderate	18 (3.9)	8 (3.9)	10 (4.1)	32 (6.0)
Severe	10 (2.2)	6 (2.9)	4 (1.7)	22 (4.2)
Alcoholism				
Never	321 (71.2)	143 (70.1)	178 (72.1)	335 (63.3)
Mild	121 (26.8) ^a^	57 (27.9)	64 (25.9) ^c^	169 (31.9)
Moderate	8 (1.8) ^a^	3 (1.5)	5 (2.0)	21 (3.9)
Severe	1 (0.2)	1 (0.5)	0 (0)	4 (0.7)
Vaccination center location				
Chalco	94 (20.8)	47 (23.0)	47 (19.0)	118 (22.3)
Toluca	69 (15.3)	35 (17.2)	34 (13.7)	135 (25.5)
Zinacantepec	94 (20.8)	29 (14.2)	65 (26.3)	77 (14.6)
Ecatepec	75 (16.6)	37 (18.1)	38 (15.4)	104 (19.7)
Acolman	119 (26.4)	56 (27.5)	63 (25.5)	95 (17.9)

Data are presented as numbers (%) or median (interquartile range). *p*-value refers to Chi-square test/^d^ Kruskal–Wallis and the letter denotes the column with a statistically significant pairwise comparison exists. ^a^ Prior COVID-19 vs. seronegative. ^b^ symptomatic vs. seronegative. ^c^ symptomatic vs. seronegative. The age and weight data of 3.3% and 7.5% of the participants with prior covid-19 were not available; and 3.2% and 6.6% in seronegative group, respectively. Abbreviations: Kg, kilogram; m, meter; BMI, body mass index; HIV, human immunodeficiency virus. * Seroprevalence was categorized as prior COVID-19 (symptomatic and asymptomatic), and seronegative. ** Body mass index was categorized as normal weight (<25 Kg/m^2^), overweight (25 to 29.9 Kg/m^2^), obesity (≥30 Kg/m^2^), class I obesity (30 to 34.9 Kg/m^2^), class II obesity (35 to 39.9 Kg/m^2^), class III obesity (≥40 Kg/m^2^).

**Table 2:** Association of Obesity with SARS-CoV-2 infection.

Characteristic	Prior COVID-19 vs. Seronegative	Asymptomatic vs. Seronegative	Symptomatic vs. Seronegative	Symptomatic vs. Asymptomatic
	OR (95% CI); *p*-Value	OR (95% CI); *p*-Value	OR (95% CI); *p*-Value	OR (95% CI); *p*-Value
Unadjusted analysis				
Normal weight	Ref			
Overweight	1.29(0.93–1.80); 0.1230	1.44(0.95–2.20); 0.0850	1.16(0.76–1.76); 0.4860	0.80(0.47–1.34); 0.4010
Obesity	2.11(1.49–3.01); <0.0001	2.49(1.61–3.86); <0.0001	1.77(1.14–2.75); 0.0110	0.71(0.42–1.19); 0.1990
Class Obesity				
Class I Obesity	1.94(1.33–2.83); <0.0001	2.33(1.47–3.70); <0.0001	1.59(0.99–2.55); 0.0540	0.68(0.39–1.18); 0.1760
Class II Obesity	3.15(1.62–6.11); 0.0010	3.30(1.50–7.23); 0.0030	3.01(1.38–6.56); 0.0050	0.91(0.39–2.09); 0.8300
Class III Obesity	2.18(0.92–5.19); 0.0770	2.81(1.04–7.59); 0.0400	1.60(0.52–4.94); 0.4070	0.57(0.17–1.88); 0.3570
Adjusted analysis				
Normal weight	Ref			
Overweight	1.30(0.93–1.82); 0.1210	1.45(0.94–2.24); 0.0850	1.16(0.76–1.78); 0.4710	0.79(0.46–1.34); 0.3830
Obesity	2.18(1.51–3.16); <0.0001	2.58(1.63–4.09); <0.0001	1.88(1.18–2.98); 0.0070	0.71(0.41–1.23); 0.2320
Class Obesity				
Class I Obesity	1.99(1.35–2.95); 0.0010	2.44(1.50–3.98); <0.0001	1.64(1.002–2.69); 0.0490	0.65(0.36–1.17); 0.1580
Class II Obesity	3.02(1.54–5.91); 0.0010	3.12(1.38–7.02); 0.0060	3.02(1.37–6.62); 0.0060	0.95(0.40–2.25); 0.9100
Class III Obesity	2.46(0.99–6.10); 0.0520	3.12(1.08–8.94); 0.0340	1.82(0.56–5.88); 0.3150	0.56(0.16–1.91); 0.3570

All associations were tested using logistic regression adjusted by age, gender, smoking, and alcohol.

## Data Availability

The data set(s) supporting the results of this article are included within the article.
